# On Bony Growths Invading the Tonsils

**Published:** 1896-07

**Authors:** Alex. W. Stirling

**Affiliations:** M.D., (Hon.) M.B., C.M. (Edin.), D.P.H. (Lond.); Atlanta, Ga.


### ON BONY GROWTHS INVADING THE TONSILS.*

By ALEX. W. STIRLING, M.D., (Hon.) M. B., C.M. (Edin.), D.P.H. (Lond.),

ATLANTA, GA.

The following three cases† are, so far as I can discern, unique in the medical literature of the English language, though one case of the kind is mentioned by Schech as having been noted from the clinical, and another from the anatomical, point of view.‡ I rather think, however, that they will yet be found to be not un-

*Abstract of a paper read before the Laryngological Section of the American Medical Association at the recent meeting.

†These cases were described before the Atlanta Medical Society, on June 2d, when one of them was shown.

‡Two of these cases were seen by a committee of four of the Laryngological Section of the American Medical Association, to all of whom they were a new observation.

common. They are interesting from the standpoint of the anatomist, the general surgeon, and, above all, of the throat specialist.

The *first case* is that of a young lady of excellent family and personal history, but troubled for years by an enlarged and occasionally painful right tonsil, which had already been treated in different ways by several throat specialists elsewhere.

On examination with the finger I was able to make out a hard, immovable substance, coming from behind the tonsil forward underneath it to the level of its anterior surface, but forming practically a part of it. Its point is rounded, apparently about the eighth of an inch in diameter, but becoming broader and somewhat flattened laterally as it goes outward, backward, and slightly upwards. The finger pressed in front of it enters an angle formed by it and the inferior maxilla, and when pressed behind it enters another angle formed by its approximation to the right side of the vertebral column. Nothing of the kind can be discovered on the left side.

The *second case* is sixty-four years of age. On examining her throat I observed a slight protuberance just above and in front of, but involving, her right tonsil. The finger showed it to be nearly the same as that already described, with the following points of difference: it exists in the second case on both sides, though it is not quite so prominent upon the left; it is also higher, farther forward, and perhaps a little thinner, and with a more upward direction, in the second case.

This patient has never had the slightest trouble with her tonsils.

In one of her daughters whom I have examined this condition of the throat is not present.

The *third case* is a brother of the second, and sixty-five years old. He likewise has been free from throat affections, but on both sides he has the peculiarity under discussion. The hard substance in him is altogether in the posterior part of the tonsils, is much longer and reaches a full finger-breadth below the level of the lower border of the tonsil, is perhaps more slender than those already described, and is for about half an inch of the lower end on either side cartilaginous to the feel and movable.

These are evidently not tonsillar calculi, neither are they the result of disease; they can only be congenital peculiarities, having, however, a distinct interest in cases of disease in their neighborhood.

If they came from the lower jaw, to which they closely approximate, they would move along with it, but this they do not do.

They do not come from the bodies of the vertebræ, because the finger can exclude these. The pterygoid plates are too far forward, and the spines on the posterior extremities of the wings of the sphenoid are also too far distant. By a process of exclusion the transverse processes of the vertebræ and the styloid processes alone remain.

The tonsil is situated on the level of the upper part of the body of the axis, or of the disc between it and the atlas. The transverse process of the axis is small, and does not move on motion of the head: the bones under consideration do move along with the head, and come into much greater and visible prominence when the head is moved toward the side opposite to that under examination. We are therefore reduced to the atlas, which is, as regards relation of the head, a part of it, and the styloid process. The only way of which I can think to distinguish between these is that the styloid process would move with the head on nodding and the atlas would not; and as these bones do so move, I conclude that we have to deal with elongated styloid processes somewhat diverted perhaps from their normal direction.

The only case in which the question of *treatment* arises is the first. The tonsil is here troublesome, but I prefer to leave the bone untouched, and rather fear to remove any of the tonsil itself, because of the likelihood that it might, on contraction, become tightly stretched over the end of the bone, and adherent to it, or that the bone might ulcerate through.

---

LET your most trusted assistant administer the anesthetic. Any intelligent person is able, with a little direction, to assist at the wound, but it requires skill and experience to anesthetize thoroughly and safely.—*Internat. Journal Surgery.*

